# Awareness of hypertension guidelines and the diagnosis and evaluation of hypertension by primary care physicians in Nigeria

**DOI:** 10.5830/CVJA-2016-048

**Published:** 2017

**Authors:** OK Ale, RW Braimoh

**Affiliations:** Department of Medicine, Faculty of Clinical Sciences, University of Lagos/Lagos University Teaching Hospital, Lagos, Nigeria; Department of Medicine, Faculty of Clinical Sciences, University of Lagos/Lagos University Teaching Hospital, Lagos, Nigeria

**Keywords:** hypertension, guidelines, diagnosis, work up, primarycare physicians, Nigeria

## Abstract

**Background:**

The availability of numerous hypertension guidelines seems not to have impacted significantly on the burden of hypertension. We evaluated awareness of hypertension guidelines among primary-care physicians (PCPs) in Nigeria and its relationship to hypertension diagnosis and work up.

**Methods:**

Anonymous self-administered questionnaires were filled in by PCPs categorised into two groups: hypertension guideline aware (GA) and unaware (GU).

**Results:**

The 403 participating PCPs had a mean age and experience of 40 ± 11.34 and 14 ± 11.10 years, respectively, with 46.7% (n = 188) of them being GA. Out of the 19 questions assessed, GA and GU PCPs performed better in seven and two questions, respectively, while the two subgroups had a similar performance in 10 questions. The performance of the PCPs in government and private practice was similar.

**Conclusions:**

There is a gap between guideline recommendations and hypertension care in Nigeria that is further widened by PCPs’ unawareness of the guidelines. Popularising hypertension guidelines among PCPs may significantly improve hypertension care and reduce the burden of disease.

## Background

Hypertension is a major public health challenge, with increasing prevalence worldwide.^1^ It is the leading cardiovascular (CV) risk factor for morbidity and mortality and the largest contributor to the global burden of disease.^2^,^3^ Approximately 40% of adults aged 25 years and older worldwide had hypertension in 2008, with Africa and the Americas having the highest (46%) and lowest (35%) prevalence, respectively.^4^ The estimated prevalence of hypertension in Nigeria is 29.5%.^5^

Undiagnosed, uncontrolled and inappropriately managed hypertension is associated with a high risk for morbidity and mortality from potentially preventable complications such as stroke, and kidney and heart diseases.^2^,^3^,^6^ However, evidence from clinical and epidemiological research has provided huge capabilities for lowering blood pressure in almost every person with hypertension.^1^ This evidence has been collated, evaluated and summarised into hypertension guidelines to assist physicians in selecting the best hypertension-management strategies, taking into account the impact on outcome, as well as the risk–benefit ratio of particular diagnostic or therapeutic means. Few of these guidelines are indigenous to sub-Saharan Africa (SSA) despite the huge burden of hypertension in this region, and many of the available guidelines do not factor the peculiarities of SSA into their recommendations.

Although it is important to consider the science of medicine for the treatment of hypertension, particular consideration should be given to cost-effectiveness and affordability because many countries in SSA have severe resource constraints.^7^ A good combination of science, cost-effectiveness and affordability is provided by the International Forum for Hypertension control and prevention in Africa (IFHA) recommendations for the prevention, diagnosis and management of hypertension and cardiovascular risk factors in sub-Saharan Africa.^7^

The presence of hypertension guidelines seems not to have significantly impacted on hypertension control in SSA, with the burden of hypertension increasing. Its epidemiology is generally characterised by low levels of awareness, poor treatment, poor blood pressure control and a high burden of hypertensionrelated complications.^2^,^3^,8

Hypertension is the commonest condition in the primary-care setting, and in many countries it is almost entirely managed by primary-care physicians (PCPs).^5^,^9^ It has been suggested that the detection and treatment of hypertension in the primary healthcare setting in SSA is poor.^7^ This is similar to the unsatisfactory management of hypertension and cardiovascular risk factors reported in various parts of the world.^10^-^12^

Limited knowledge of hypertension by healthcare professionals, among other factors, has been identified as being responsible for poor hypertension control in SSA.^3^ This makes it worthwhile to investigate the contribution of PCPs to the burden of undiagnosed and inadequately/inappropriately managed hypertension in Nigeria. Our aim was to evaluate awareness of hypertension guidelines among PCPs in Lagos, Nigeria and its effect on their diagnostic approach to hypertension. We also sought to determine the relationship between the type of practice, namely private or government, and hypertension diagnosis and work up.

## Methods

Four hundred and three Lagos-based PCPs (general practitioners) attending continuing medical education programmes were categorised into two groups: hypertension guideline aware and guideline unaware. Hypertension guideline awareness status was defined by a ‘yes’ or ‘no’ answer to the question: ‘are you aware of hypertension guidelines?’ Physicians with speciality training in internal medicine were excluded from the study.

Ethical clearance was obtained from the ethics and research committee of the Lagos University Teaching Hospital. Consent of each participant was obtained.

Anonymous self-administered questionnaires consisting of 19 open-ended and closed questions on hypertension diagnosis and work up were used. The closed questions had either yes/ no or Likert-type scale responses. The study questionnaire was in four main domains: (1) type of practice – private versus government and number of patients seen; (2) hypertension detection – frequency of blood pressure checks in patients, resting before blood pressure measurement, number of blood pressure readings, blood pressure threshold levels; (3) clinical evaluation – personal history of diabetes mellitus, alcohol and tobacco habits, family history of diabetes and hypertension, evaluation for obesity, and blood pressure measurement; and (4) laboratory/ancillary evaluation – urinalysis, serum electrolytes and creatinine, blood glucose, lipogram, electrocardiogram and fundoscopy. An additional question on hypertension being a major public health problem was included.

## Statistical analysis

Likert-type scale responses were transformed into dichotomous responses of appropriate/yes (‘always done’ and ‘often or usually done’) and inappropriate/no (‘sometimes done’, ‘occasionally done’ and ‘rarely or never done’) practice/behaviour. Another Likert-like scale (strongly agree, agree, neutral/undecided, disagree and strongly disagree) response to the statement ‘uncomplicated hypertension is usually asymptomatic’ was transformed into yes (strongly agree, agree) and no (neutral/undecided, disagree and strongly disagree). Definitions were adopted for binary outcomes based on the IFHA recommendations for prevention, diagnosis and management of hypertension and cardiovascular risk factors in sub-Saharan Africa. 7

All statistical data were analysed using the Statistical Package for Social Sciences (SPSS, version 16.0). Descriptive statistics were used to report the findings. Categorical and continuous variables were expressed as proportions and means ± SD respectively. The statistical significance of variables was tested using the chi-squared test for categorical variables and Student’s t-test for continuous variables. All tests were two-sided and values were considered statistically significant if p < 0.05.

## Results

Data from 413 PCPs with a mean age of 40 ± 11.34 years and a mean post-registration experience of 14.30 ± 11.00 years were analysed. Guideline awareness among the cohort was 46.7% (n = 188). Tables 1 and 2 show the basic characteristics of the PCPs according to their awareness of hypertension guidelines and the type of practice, respectively. The guideline-aware (GA) physicians were younger than the guideline-unaware (GU) physicians (p < 0.05). The GA and GU physicians were similar in terms of gender, experience and patient load (p < 0.05). Hypertension was considered a major public health problem by 95.1% (n = 369) of the physicians.

**Table 1:** Basic characteristics of the respondents according to their awareness of guidelines

		*Awareness of guidelines*		
	All	Yes (GA)	No (GU)	
Variable (n)	n (%) mean ± SD	n (%) mean ± SD	n (%) mean ± SD	GA vs GU χ^2^/p-value
No of physicians	403 (100)	188 (46.7)	215 (53.3)	
Age (397)	40.0 ± 11.3	38.5 ± 9.6	41.4 ± 12.6	0.01
Gender (403)				0.99/0.32
Male	249 (61.8)	121 (64.4)	128 (59.5)	
Female	154 (38.2)	67 (35.6)	87(40.3)	
Years post registration (403)	14.3 ± 11.1	13.4 ± 9.9	15.1 ± 12.0	0.12
No of patients seen per day (403)	17.4 ± 14.3	17.5 ± 11.6	18.3 ± 16.2	0.58
No of hypertensive patients seen per day (396)	4.4 ± 3.5	4.1 ± 3.3	4.6 ± 3.6	0.21
Type of practice (403)				5.95/0.015
Private (269)	269 (66.7)	137 (72.9)	132 (61.4)	
Government (134)	134 (33.3)	51 (27.1)	83 (38.6)	
Consider hypertension a public health challenge (388)	369 (95.1)	164 (94.8)	205 (95.3)	0.06/0.80

GA, guideline aware; GU, guideline unaware.

**Table 2:** Basic characteristics of the respondents according to the type of practice

	Type of practice			
	All	Private	Government	
Variable (n)	n (%) mean ± SD	n (%) mean ± SD	n (%) mean ± SD	Private vs government χ2/p-value
No of physicians	403 (100)	269 (66.7)	134 (33.3)	
Age (397)	40.0 ± 11.3	42.6 ± 11.9	35.0 ± 7.9	< 0.001
Gender (403)				20.47/ < 0.001
Male	249 (61.8)	187(69.5)	62(46.3)	
Female	154 (38.2)	82(30.5)	72(53.7)	
Years post registration (403)	14.3 ± 11.1	16.9 ±11.4	9.2 ± 8.5	< 0.001
No of patients seen per day (403)	17.4 ± 14.3	15.8 ± 10.8	22.2 ± 18.7	< 0.001
No of hypertensive patients seen per day (396)	4.4 ± 3.5	3.3 ± 2.3	6.5 ± 4.3	< 0.001
Awareness of guidelines (403)	188 (46.7)	137 (50.9)	51 (38.1)	5.95/0.015

Table 3 shows hypertension knowledge, diagnosis and work up by the PCPs according to their awareness of hypertension guidelines. Out of the 19 questions asked, the GA PCPs performed better than the GU physicians in seven, the GU PCPs performed better than the GA physicians in two, and the two groups had a similar performance in the remaining 10 questions. The practice of routinely checking blood pressure of all adult patients in consultation was independent of whether or not the physicians considered hypertension a major public health challenge (χ2 = 0.07, p = 0.8).

**Table 3:** Hypertension knowledge, diagnosis and work up by the respondents according to their awareness of guidelines

		Awareness of guidelines		
	All	Yes (GA)	No (GU)	
Variable (n)	n (%)	n (%)	n (%)	GA vs GU χ^2^/p-value
Correct BP threshold for hypertension diagnosis (403)	301 (74.7)	158 (84)	143 (66.5)	76.3/ < 0.001
Routinely checked BP in practice (392)	273 (69.6)	144 (80.9)	129 (60.3)	19.5/< 0.001
Allows short rest before measuring BP (390)	103 (26.4)	51 (28.3)	52 (24.8)	0.64/0.425
Take ≥ two BP readings before diagnosing hypertension (403)	398 (98.8)	188 (100)	210 (97.7)	0.064*
Measures BP in both arms during first visit (390)	63 (16.2)	36 (20)	27 (12.9)	3.65/0.056
Agreed uncomplicated hypertension is asymptomatic (403)	281 (69.7)	111 (59)	170 (79)	< 0.001*
FH of hypertension (398)	349 (87.7)	173 (92)	176 (83.8)	6.2/0.014
FH of DM ( 403)	305 (75.7)	163 (86.7)	142 (66.6)	< 0.001*
PH of DM ( 400)	312 (78)	161 (87)	151 (70)	< 0.001*
Obesity evaluation ( 400)	183 (45.8)	93 (50.3)	90 (41.9)	2.8/0.092
Alcohol history (403)	297 (73.7)	137 (72.9)	160 (74.4)	0.12/0.73
Tobacco history (398)	297(74.6)	142 (75.5)	155 (73.8)	0.16/0.69
Physical activity evaluation (383)	251 (65.5)	128 (71.9)	123 (60)	6.0/0.014
Urinalysis (403)	324 (80.4)	163 (86.7)	161 (74.9)	3.9/0.003
Blood glucose (398)	248 (62.3)	124 (66)	124 (59)	0.18*
EUCr (399)	245 (61.4)	120 (65.2)	125 (58.1)	2.1/0.15
Lipogram (403)	166 (41.2)	73 (38.8)	93 (43.3)	0.8/0.37
Fundoscopy (400)	21 (5.3)	0 (0)	21 (9.8)	< 0.001*
Electrocardiography (398)	204 (51.3)	101 (53.7)	103 (49)	0.87/0.35

GA, guideline aware; GU, guideline unaware; BP, blood pressure; FH, family history; PH, personal history; DM, diabetes mellitus; EUCr, serum electrolytes and creatinine; *Fishers exact test.

Table 4 shows hypertension knowledge, diagnosis and work up by the PCPs according to the type of practice. One-third (n = 134) of respondents were in government practice. PCPs in private practice were older, more likely to be male, had more years of experience, saw fewer patients, and had a higher prevalence of hypertension guideline awareness (p < 0.05). Out of the 19 questions asked, physicians in private practice performed better in three, those in government practice also performed better in three, and the performance of the two groups in the remaining 13 questions was similar.

**Table 4:** Hypertension knowledge, diagnosis and work up by the respondents according to their type of practice

	Type of practice					
Variable (n)	All n (%)	Private n (%)	Government n (%)	Private vs government χ^2^/ p-value
Correct BP threshold for hypertension diagnosis (403)	301 (74.7)	208 (77.3)	93 (69.4)	2.97/0.085
Routinely check BP in practice (392)	273 (69.6)	201 (76.4)	72 (55.8)	17.39/< 0.001
Allows short rest before measuring BP (390)	103 (26.4)	74 (28.4)	29 (22.5)	1.53/0.22
Take ≥ two BP readings before diagnosing hypertension (403)	398 (98.8)	264 (98.1)	134 (100)	0.175*
Measures BP in both arms during first visit (390)	63 (16.2)	44 (16.7)	19 (15.1)	0.16/0.69
Agrees uncomplicated hypertension is asymptomatic (403)	281 (69.7)	177 (65.8)	104 (77.6)	5.9/0.015
FH of hypertension (398)	349 (87.7)	229 (86.7)	120 (89.6)	0.65/0.42
FH of DM ( 403)	305 (75.7)	212 (78.8)	93 (69.4)	4.30/0.04
PH of DM ( 400)	312 (78)	201 (75.6)	111 (82.8)	2.75/0.1
Obesity evaluation ( 400)	183 (45.8)	122 (45.4)	61 (45.5)	0.052/0.82
Alcohol history (403)	297 (73.7)	201 (74.7)	96 (71.6)	0.44/0.51
Tobacco history (398)	297 (74.6)	211 (79.9)	86 (64.2)	11.64/0.001
Physical activity (383)	251 (65.5)	180 (70.9)	71 (53)	9.49/0.002
Urinalysis (403)	324 (80.4)	242 (90)	82 (61.2)	47/< 0.001
Blood glucose (398)	248 (62.3)	164 (62.1)	84 (62.7)	0.01/0.91
EUCr (399)	245 (61.4)	153 (57.7)	92 (68.7)	4.48/0.03
Lipogram (403)	166 (41.2)	104 (38.7)	62 (46.3)	2.14/0.14
Fundoscopy (400)	21 95.3)	17(6.3)	4 (3.1)	1.89/0.17
Electrocardiography (398)	204 (51.3)	136 (51.5)	68 (50.7)	0.02/0.89

BP, blood pressure; FH, family history; PH, personal history; DM, diabetes mellitus; EUCr, serum electrolytes and creatinine; *Fisher’s exact test.

## Discussion

Identification of deficiencies in the approach of physicians to the prevention, diagnosis and management of hypertension is a prerequisite for planning interventions targeted towards hypertension control. Hypertension guidelines summarise evidence-based best practices aimed at improving hypertension diagnosis, evaluation, treatment and control. Knowledge of and adherence to guidelines by care givers is imperative for effective hypertension control. This will also help reduce the high risk of cardiovascular morbidity and mortality from the potentially preventable complications of hypertension, such as heart failure, kidney disease and stroke.^13^

Less than half of the respondents in this study (46.7%) were aware of the hypertension guidelines. This proportion is unsatisfactory but smaller than the 68.8% recorded for PCPs in South Africa.^13^ This suggests that hypertension management by most of the PCPs in our study may not be evidence based. This is disquieting as it suggests that most hypertensive patients in Nigeria may not be benefiting from diagnostic and therapeutic advances in hypertension management since most individuals with hypertension are managed by PCPs.^9^ This survey finding represents a potential cause for concern as it may be responsible for the high burden of hypertension-related complications in Nigeria.^2^,^8^ However, the paucity of hypertension guidelines indigenous to SSA may be a reason for the above findings.

Hypertension rarely causes symptoms in the early stages and in many people it goes undiagnosed.^4^ The fact that over two-thirds of hypertensive individuals in Nigeria are unaware of their hypertensive status makes proper surveillance for the detection of hypertensive individuals imperative for good hypertension control.^2^ This underlies the IFHA recommendation of blood pressure checks on all adult healthcare seekers at every encounter with healthcare providers.^7^

Two-thirds (69.9%) of the PCPs in this study routinely checked the blood pressure of patients in consultation. A similar proportion of the PCPs in this study (69.7%) also agreed that uncomplicated hypertension is usually asymptomatic. This however contrasts sharply with the high proportion (95.1%) of physicians who considered hypertension a major public health challenge. These findings suggest that the knowledge of the enormity of the challenge posed by hypertension may have been overridden by their inadequate knowledge of the symptomatology of hypertension. The effect of this is reflected in the lower proportion of PCPs who routinely checked the blood pressure of their adult clients in consultation.

Running clinics that are very busy may also have contributed to the discordance between knowledge of the enormity of the hypertension burden and performing routine blood pressure checks on all adult patients. Our finding is however similar to that of an earlier survey by Ajuluchukwu et al.^11^ of general practitioners in Nigeria where 70% of the PCPs routinely checked the blood pressure of their patients in consultation. It is however lower than the 80.5 and 87% reported for Cameroon- and Australia-based PCPs.^14^,^15^ This practice may largely underlie the high burden of undiagnosed hypertension and hypertensive target-organ damage in Nigeria, although factors related to patient and healthcare systems such as poor health-seeking behaviour and the use of alternative medical practitioners may also be contributory.^2^,^8^ Symptoms of target-organ damage is what often brings patients with hypertension to healthcare facilities in Nigeria.^2^

The casual measurement of blood pressure varies widely, hence certain measures are recommended to improve its reliability.^7^,^16^ This includes making the patients sit comfortably for some minutes before blood pressure measurement is carried out, the measurement of blood pressure on both arms during the patient’s first visit, and subsequently choosing the arm with higher blood pressure as the reference.^7^,^12^

Only 26.4% of all respondents allowed a rest of 10 minutes or more, recommended by the IFHA guidelines.^7^ This recommended period appears to be too long for it to be practicable in routine clinical practice, hence the small proportion of respondents adhering to it. A shorter duration of rest, the five minutes recommended by the American JNC 7 guidelines,^17^ appears more practicable in day-to-day clinical practice considering the workload in primary healthcare facilities.

The small proportion of respondents (16.2%) who measured blood pressure on both arms during a patient’s first visit may be due to high patient load or outright ignorance of this recommended practice. This contrasted sharply with the 55.1% of India-based PCPs who recorded blood pressure on both arms.^18^

PCPs may miss the clues for secondary hypertension by initial measurement of blood pressure on only one arm. Subjects with hypertension may be wrongly labelled as normotensive, and uncontrolled hypertension assessed as being controlled by the inadvertent use of the arm with a lower blood pressure value for evaluation. The practice of not identifying the arm with higher blood pressure and using it as the reference may also be contributory to the high burden of undiagnosed hypertension, uncontrolled hypertension and hypertensive target-organ damage in Nigeria.^2^,^7^,^8^ The above underscores a comment by Kaplan that the measurement of blood pressure is the clinical procedure of greatest importance that is performed in the sloppiest manner.^19^

Evaluation of the total cardiovascular risk of hypertensive individuals is recommended by the guidelines. Apart from assisting in prognostication, modification of some of these risk factors is associated with blood pressure reduction.^7^,^17^,^20^ On the other hand, failure to adhere to risk-factor modification, such as weight reduction for obese subjects, may result in resistant hypertension.^7^,^17^,^20^ A large majority of the PCPs clinically evaluated their patients for these risk factors, with the exception of obesity, which was performed by less than half of the PCPs. Not paying adequate attention to obesity in individuals with hypertension may be contributory to the high burden of uncontrolled hypertension reported globally.^2^,^11^,^12^

Another evaluation carried out routinely by a minority (41.2%) of the PCPs was lipograms. This may be predicated on the belief that it is not an important investigation in sub-Saharan African blacks because of low levels of cholesterol.^21^ However recent studies have not only shown that lipid abnormalities are common in Nigerians newly presenting with hypertension, but also that these abnormalities worsen with the severity of hypertension.^22^,^23^

A very small proportion (5.3%) of respondents examined the optic fundus of their hypertensive patients. This is lower than the 18.9 and 56.6% reported for PCPs in Italy and Slovenia, respectively.^24^,^25^ It is however instructive to note that optic fundus examination was the least-frequently performed element of the minimal hypertension diagnostic procedures, not only in the current study, but also in the Italian and Slovenian studies.^24^,^25^ Likely reasons for this may include inadequate medical consultation time and dearth of skills and/or equipment for optic fundus examination.

Though the approach of the practitioners in private and government practice to the evaluation of hypertension was heterogeneous, their overall performance was similar. Out of the 19 questions asked (excluding questions on awareness of guidelines) the PCPs in private practice performed better than those in government practice in their responses to three questions, and vice versa to three other questions. The performance of the two groups in the remaining 13 questions was similar. The reason for this similarity in the overall performance by these two groups is not apparent in this study, but we dared to postulate that it may have been due to the effect of PCPs in private practice having more time to read and adhere to guidelines being offset by the effect of better exposure to continuing professional education (practical and theoretical) by PCPs in government practice.

As expected, the PCPs in the guideline-aware group performed better than those in the unaware group (seven out of 19 responses vs two out of 19 responses). This shows that hypertension guideline awareness is associated with better hypertension care and that awareness of these guidelines should be promoted among PCPs. In spite of these findings, the general performance of the guideline-aware PCPs was unsatisfactory. This may have been due to them not being conversant with the content of the hypertension guidelines despite being aware of the guidelines.

This scenario was reported among South Africa-based PCPs by Parker et al. where 68.8% of the PCPs were aware of hypertension guidelines, but only 18.2% of the guideline-aware PCPs were conversant with the content thereof.^13^ A preference for the use of personal experience that is not evidenced based over evidence-based recommendations contained in guidelines has been documented among PCPs in Croatia. A similar scenario may have played out in our cohort of PCPs. Inadequate time for medical consultation may also be contributory to the suboptimal general performance of guideline-aware PCPs.

Limitations of this study include the use of a self-administered questionnaire, which is limited by the varying abilities of the participants to recall. This study evaluated the knowledge of the PCPs, which may not represent their actual practices. Obtaining data from medical records would have given an excellent picture of what these PCPs actually do.

## Conclusion

Considering the enormity of issues related to hypertension in terms of the large segment of the population involved, the commonness of the condition in primary care (one in every four patients seen in this study), and the attendant morbidity and mortality rates, the performance of the cohort in in this study, irrespective of their guideline-awareness status, was unsatisfactory and poses significant challenges to hypertension care in Nigeria. The findings of this study suggest inadequate assessment of target-organ damage and patient risk stratification, with consequent poor global cardiovascular risk management, contrary to guideline recommendations.^7^,^10^,^20^

However, the fact that awareness of hypertension guidelines by PCPs is associated with improved hypertension care, as shown in this study, makes continuing professional education of general practitioners in evidence-based hypertension care, as expounded in hypertension guidelines, imperative in bridging the gap between the current reality and the desired in hypertension care.
